# Resolution of anaemia in a cohort of HIV-infected patients with a high prevalence and incidence of tuberculosis receiving antiretroviral therapy in South Africa

**DOI:** 10.1186/s12879-014-0702-1

**Published:** 2014-12-21

**Authors:** Andrew D Kerkhoff, Robin Wood, Frank G Cobelens, Ankur Gupta-Wright, Linda-Gail Bekker, Stephen D Lawn

**Affiliations:** George Washington University School of Medicine and Health Sciences, 2300 I St, NW, 20037 Washington, DC USA; Department of Global Health, Academic Medical Center, Amsterdam Institute for Global Health and Development, University of Amsterdam, Amsterdam, The Netherlands; The Desmond Tutu HIV Centre, Institute of Infectious Disease and Molecular Medicine, Faculty of Health Sciences, University of Cape Town, Cape Town, South Africa; KNCV Tuberculosis Foundation, The Hague, The Netherlands; Department of Clinical Research, Faculty of Infectious and Tropical Diseases, London School of Hygiene and Tropical Medicine, London, UK

**Keywords:** HIV, Tuberculosis, Africa, Anaemia, Haemoglobin, Antiretroviral

## Abstract

**Background:**

Anaemia is frequently associated with both HIV-infection and HIV-related tuberculosis (TB) in antiretroviral therapy (ART)-naïve patients in sub-Saharan Africa and is strongly associated with poor prognosis. However, the effect of ART on the resolution of anaemia in patient cohorts with a high prevalence and incidence of tuberculosis is incompletely defined and the impact of TB episodes on haemoglobin recovery has not previously been reported. We therefore examined these issues using data from a well-characterised cohort of patients initiating ART in South Africa.

**Methods:**

Prospectively collected clinical and haematological data were retrospectively analysed from patients receiving ART in a South African township ART service. TB diagnoses and time-updated haemoglobin concentrations, CD4 counts and HIV viral loads were recorded. Anaemia severity was classified according to WHO criteria. Multivariable logistic regression analysis was used to determine factors independently associated with anaemia after 12 months of ART.

**Results:**

Of 1,140 patients with baseline haemoglobin levels, 814 were alive in care and had repeat values available after 12 months of ART. The majority of patients were female (73%), the median CD4 count was 104 cells/uL and 30.5% had a TB diagnosis in the first year of ART. At baseline, anaemia (any severity) was present in 574 (70.5%) patients and was moderate/severe in 346 (42.5%). After 12 months of ART, 218 (26.8%) patients had anaemia of any severity and just 67 (8.2%) patients had moderate/severe anaemia. Independent predictors of anaemia after 12 months of ART included greater severity of anaemia at baseline, time-updated erythrocyte microcytosis and receipt of an AZT-containing regimen. In contrast, prevalent and/or incident TB, gender and baseline and time-updated CD4 cell count and viral load measurements were not independent predictors.

**Conclusions:**

Although anaemia was very common among ART-naive patients, the anaemia resolved during the first year of ART in a large majority of patients regardless of TB status without routine use of additional interventions. However, approximately one-quarter of patients remained anaemic after one year of ART and may require additional investigations and/or interventions.

**Electronic supplementary material:**

The online version of this article (doi:10.1186/s12879-014-0702-1) contains supplementary material, which is available to authorized users.

## Background

Anaemia is the most common haematological manifestation of HIV disease and is frequent among antiretroviral therapy (ART)-naïve patients in sub-Saharan Africa, with a prevalence ranging from 45- 87% [1]-[5]. HIV-related anaemia is associated with a decreased quality of life [6]-[8], accelerated HIV disease progression [9],[10], increased risk of virological failure [11]-[13] and decreased survival [9],[14]-[17] .

The mechanisms contributing to HIV-related anaemia are complex. HIV itself may result in an up-regulation of cytokines and hepcidin causing anaemia through inhibition of mucosal uptake of dietary iron and sequestration of iron in bone marrow macrophages [18]. HIV may also cause dysregulated erythropoiesis through direct viral infection of bone marrow progenitor cells, although this remains controversial [19]-[23]. Tuberculosis (TB) is the commonest opportunistic infection among HIV-infected patients in sub-Saharan Africa and is likely an important cause of HIV-related anaemia, where anaemia occurs in up to 90% of patients with HIV-associated TB [24]-[27]. Similar to HIV, TB may also cause an anaemia of chronic disease through upregulation of proinflammatory cytokines [28],[29]. Additionally, dissemination of TB to the gastro-intestinal tract mucosa may result in iron deficiency anaemia [30],[31], while bone marrow involvement may cause impairment of all hematopoietic cell lines [32]-[34]. In sub-Saharan Africa additional factors may contribute to anaemia in people living with HIV, including nutritional deficiencies of iron, folate and vitamin B12 as well as co-morbidities such as malaria, helminth infections and opportunistic infections and also adverse drug effects.

Large studies from industrialized countries appear to indicate that antiretroviral therapy (ART) has strong positive effects on haemoglobin recovery [35]-[39]. In sub-Saharan Africa where the prevalence of HIV-related anaemia remains high, previous studies have shown that ART is likely associated with significant haemoglobin recovery [1],[2],[40]-[46]. However, this is complicated by a lack of an international consensus definition for anaemia, which makes comparison of previous studies from this region difficult. It is also unknown whether patients with HIV-associated TB achieve similar haemoglobin recovery as patients without TB. Therefore, we undertook a retrospective cohort analysis to characterize changes in haemoglobin concentration during the first year of ART among patients in Cape Town with a high prevalence and incidence of TB, define what proportion of patients had resolution of baseline anaemia or maintained normal haemoglobin levels after 12 months of ART and determine risk factors independently associated with anaemia after 12 months of ART.

## Methods

### Study setting

The present study is part of on-going research at the Hannan Crusaid Antiretroviral Centre in Gugulethu township, Cape Town, South Africa which has previously been described in detail [47],[48]. Patients consecutively enrolled in the ART programme were eligible for the study if they were ART naïve, at least 16 years of age, and subsequently initiated ART. All patients provided written informed consent and the study was approved by the human ethics committee of the University of Cape Town, Cape Town, South Africa.

Patients were enrolled between September 2002 and May 2006 (a time period for which very complete clinical records and time-updated TB, CD4 count and viral load data were available) and were eligible to start ART if they had either a World Health Organization (WHO) stage 4 disease or CD4 count <200 cells/uL in accordance with national ART guidelines at that time. First-line ART regimens were comprised of two nucleoside reverse transcriptase inhibitors, which included either stavudine (d4T) or zidovudine (AZT) with lamivudine (3TC) and a non-nucleoside reverse transcriptase inhibitor (predominantly efavirenz (EFV)). All patients received daily trimethoprim-sulphamethoxazole prophylaxis. At the time, national ART guidelines gave no recommendations for the management of HIV-related anaemia and therefore no interventions for anaemia were routinely implemented.

Patients were referred for ART initiation from primary care clinics, TB clinics and antenatal clinics and received routine clinical review at a screening visit, ART initiation, after 4, 8 and 16 weeks of ART, and thereafter at least every 4 months. Haemoglobin concentrations as well as mean corpuscular volumes (MCV), blood CD4 cell counts and plasma HIV viral load levels were done prior to ART initiation and at least every 4 months thereafter.

### TB screening and diagnosis

Several different investigations were available for the diagnosis of TB either prior to ART initiation or during ART follow-up and included: sputum smear fluorescence microscopy, automated liquid culture of sputum using mycobacterial growth indicator tubes (MGIT 960, Becton Dickinson, Sparks, Maryland, USA), chest radiology, ultrasonography and fine needle lymph node aspiration (FNA) cytology. Culture-positive specimens were speciated using polymerase chain reaction (PCR). A large majority of TB cases were culture-proven, however any culture-negative TB diagnosis was defined by strong clinical or histological evidence of active TB, radiological abnormalities consistent with active TB and the clinician’s decision to treat with a full course of anti-TB therapy. Patients diagnosed with TB were given a rifampicin-based anti-tuberculosis regimen as part of a direct observed treatment short-course (DOTS) strategy.

### Definitions

Anaemia severity was classified according to WHO criteria [49]: no anaemia (haemoglobin concentration ≥13.0 g/dL for males, ≥12.0 g/dL for females), mild anaemia (11.0-12.9 g/dL for males, 11.0-11.9 g/dL for females), moderate anaemia (8.0-10.9 g/dL for males and females) or severe anaemia (<8.0 g/dL for males and females). Patients without anaemia at the time of ART initiation but who had anaemia (of any severity) after 12 months of ART were defined as having ‘incident anaemia’. ‘Persistent anaemia’ was defined as the presence of anaemia (of any severity) both prior to ART initiation and after 12 months of ART. A ‘baseline haemoglobin’ was defined as a haemoglobin concentration within 90 days of ART initiation for those without a known TB diagnosis prior to ART initiation (no prevalent TB), and within 30 days for those with prevalent TB, as anti-TB therapy is known to be associated with rapid changes in haemoglobin concentration [50]-[52].

Patients who had previously been diagnosed with TB and completed anti-TB treatment prior to enrolment in the ART cohort defined those with a ‘past history of TB’. ‘Referred TB’ was defined as those with a known TB diagnosis who were referred from TB clinics to the ART service for initiation of ART and who were currently taking TB treatment at the time of starting ART. Any new clinical episode of TB that was diagnosed in the ART service around the time of ART initiation or at any point within the first year of starting ART was defined as “TB in the first year of ART”.

### Data analysis

Data were analysed using Stata version 12.0 (College Station, Texas, USA). The main analysis was restricted to those who had haemoglobin results available at the time of ART initiation and also at 12 months after ART initiation. Time-updated intervals were defined by laboratory measurements at ART initiation (baseline) and at 4, 8 and 12 months of ART. Chi-squared tests, Wilcoxin rank-sum, Kruskal-Wallis tests and t-tests were used when appropriate. Multivariable logistic regression analysis was used to determine factors independently associated with anaemia (any severity) after 12 months of ART; a priori risk factors (gender and AZT-exposure) and variables meeting the predefined cutoff of P ≤ 0.1 in the univariable model were included in the multivariable model. Sensitivity analyses were undertaken to determine if haemoglobin recovery was affected by the exclusion of patients who died or were lost-to-follow-up in the first year of ART, where it was assumed that such patients had no haemoglobin recovery while receiving ART (median improvement of zero). All statistical tests were two-sided at a value of 0.05.

## Results

### Baseline patient characteristics

**Figure 1:**
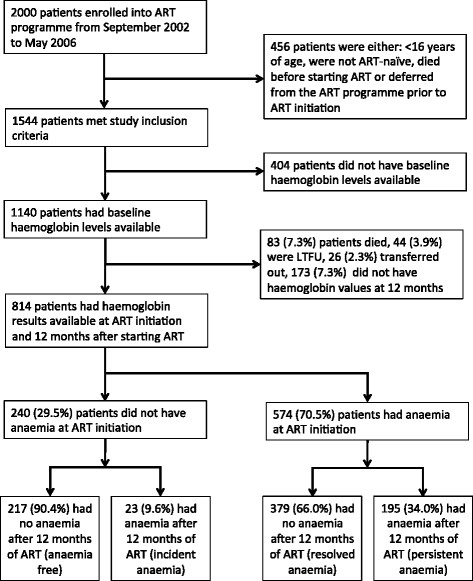
**Flow diagram showing the number of patients included in the analysis and the prevalence of anaemia.**

Of 1,544 patients meeting study inclusion criteria, 1140 had baseline haemoglobin levels available. Haemoglobin levels after 12 months of ART were not available for 326 patients due to death, loss to follow-up, transfer-out or repeat testing not being done and they were therefore excluded (Figure 1). The remaining 814 patients were included in the main analysis (Figure 1).

Patients were predominantly female and had a median age of 33 years (IQR, 29–39). They also tended to have advanced HIV disease, with a median CD4 count of 104 cells/μL (IQR, 50–156) and a median viral load of 4.8 log copies/mL (IQR, 4.4-5.2) (Table 1). There were 168 patients (20.6%) who received AZT at any point during the first year of ART, of whom 59 (7.2%) received an AZT-containing regimen continuously, 22 (2.7%) switched AZT to another drug and 87 (10.7%) switched another drug to AZT. Among the 22 patients who switched from an AZT-containing regimen, severe anaemia was cited as the reason for the drug switch in only 2 patients.

**Table 1:** **Baseline patient characteristics (n = 814)**

	WHO anaemia severity classification at ART initiation					
	All (n = 814)	None (29.5%; n = 240)	Mild (28.0%; n = 228)	Moderate(38.1%; n = 310)	Severe (4.4%; n = 36)	P-value
**Age (at baseline), median (IQR)**	33 (29–39)	34 (29–40)	33 (29–40)	32 (28–37)	31 (27–37)	0.008
**Gender**						
Male	224 (27.5)	102 (42.5)	87 (38.2)	30 (9.7)	5 (13.9)	<0.001
Female	590 (72.5)	138 (57.5)	141 (61.8)	280 (90.3)	31 (86.1)	
**WHO disease stage (at baseline)**						
1 or 2	185 (22.7)	56 (23.3)	55 (24.1)	69 (22.3)	5 (13.9)	0.584
3 or 4	629 (77.3)	184 (76.7)	173 (75.9)	241 (77.7)	31 (86.1)	
**Previous history of TB treatment**						
No	422 (51.8)	109 (45.4)	126 (55.3)	162 (52.3)	15 (69.4)	0.023
Yes	392 (48.2)	131 (54.6)	102 (44.7)	148 (47.7)	11 (30.6)	
**ART regimen**						
AZT-free throughout	646 (79.4)	207 (86.3)	187 (82.0)	221 (71.3)	31 (86.1)	<0.001
Any AZT exposure	168 (20.6)	33 (13.8)	41 (18.0)	89 (28.7)	5 (13.9)	
**CD4 cell count (cells/μL)a** ^**a**^						
Median, IQR	104 (50–156)	113 (56–164)	117 (63–169)	93 (44–146)	85 (31–125)	0.002
<100	383 (47.2)	109 (45.6)	90 (39.7)	164 (53.1)	20 (55.6)	0.013
≥100	428 (52.8)	130 (54.4)	137 (60.4)	145 (46.9)	16 (44.4)	
**HIV viral load (log copies/mL), median (IQR)** ^**b**^	4.8 (4.4-5.2)	4.8 (4.3-5.1)	4.8 (4.4-5.2)	4.9 (4.5-5.3)	5.0 (4.7-5.3)	0.002
**MCV (fL)** ^**c**^						
Median, IQR	90 (86–95)	92 (88–96)	90 (87–94)	89 (84–94)	83 (75–95)	<0.001
<80	56 (7.0)	5 (2.1)	9 (4.0)	28 (9.2)	14 (38.9)	<0.001
80-100	671 (83.7)	205 (87.0)	200 (88.5)	250 (82.2)	16 (44.4)	
>100	75 (9.4)	26 (11.0)	17 (7.5)	26 (8.6)	6 (16.7)	
**TB disease status**						
TB free in first year of ART	566 (69.5)	180 (75.0)	160 (70.2)	204 (65.8)	22 (61.1)	0.148
Referred TB	131 (16.1)	34 (14.2)	38 (16.7)	54 (17.4)	5 (13.9)	
TB in first year of ART	117 (14.4)	26 (10.8)	30 (13.2)	52 (16.8)	9 (25.0)	

^**a**^3 missing CD4 cell count results, ^b^5 missing HIV viral load results, ^c^12 missing MCV results.

Abbreviations: ART; antiretroviral therapy; AZT; zidovudine; IQR, interquartile range; MCV, mean corpuscular volume; TB, tuberculosis; WHO, World Health Organization. All values are proportions (%) unless otherwise stated.

The baseline characteristics of patients known to be alive at 12 months but for whom 12 month haemoglobin level results were missing (n = 173) or who transferred out (n = 26) in the first year of the ART programme were similar to those included in the final analysis (**data not shown**). However, those who died (n = 83) or were lost-to-follow up (n = 44) in the first year of ART were more likely to be male, have more advanced immunosuppression and have a TB diagnosis within the first year of ART compared to those included in the final analysis **(data not shown)**. Among patients who died in the first year of ART, 54 (65.1%) had moderate or severe anaemia at baseline and 52 (62.7%) had moderate or severe anaemia at the last available measurement before death. Of 62 patients who died in the first 4 months of ART, 40 (64.2%) had moderate or severe anaemia prior to ART initiation.

### Prevalence of anaemia

At the time of ART initiation, 574 patients (prevalence 70.5%, 95% CI 67.3-73.6) had anaemia, which was classified as mild, moderate, or severe in 228 (28.0%), 310 (38.1%) and 36 (4.4%) patients, respectively. Anaemia was more severe in patients who were younger, female, had lower CD4 cell counts, higher HIV viral loads and had no medical history of TB (Table 1).

### TB diagnoses

At ART initiation, 131 (16.1%) patients were receiving anti-TB therapy and 117 (14.4%) had new TB disease diagnosed within the first year of ART (Table 1). Therefore, 248 (30.5%) patients were receiving treatment for TB at some time-point during the first year of ART.

### Time-updated anaemia severity during the first year of ART

**Figure 2:**
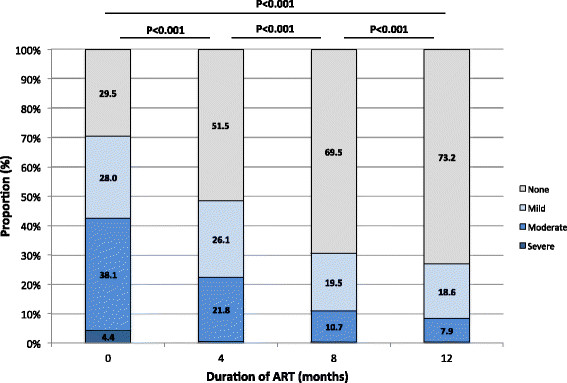
**Time-updated changes in the prevalence and severity of anaemia amongst patients during the first year of ART receipt.** Anaemia was classified according to WHO criteria. At 0, 4, 8 and 12 months of ART there were 814, 674, 714 and 814 observations, respectively.

With increasing duration of ART, the prevalence and severity of anaemia decreased (Figure 2). After 12 months of ART, 596 (73.2%) patients had normal haemoglobin levels, whereas 218 patients (26.8%) had anaemia, of which 67 patients (8.2%) had either moderate or severe anaemia. The overall prevalence of anaemia after 12 months of ART decreased by 62.0%, including an 80.6% decrease in moderate or severe anaemia. Of 199 patients who either transferred out or who were alive and in care but did not have haemoglobin measurements at 12 months, the severity and prevalence of anaemia at 4 and 8 months after ART initiation were similar to those included in the final analysis (p = 0.246 and p = 0.499, respectively).

### Persistent and incident anaemia after 12 months of ART

Among those with anaemia at the time of ART initiation (n = 574), 379 (66.0%) had normal haemoglobin levels after 12 months of ART. Of 195 (34.0%) patients with persistent anaemia after 12 months of ART, 62 (10.8%) had moderate/severe anaemia. Of 240 patients without anaemia at the time of ART initiation, 23 patients (9.6%) developed incident anaemia of which 5 (2.1%) were moderate or severe.

### Changes in haemoglobin during the first year of ART

**Figure 3:**
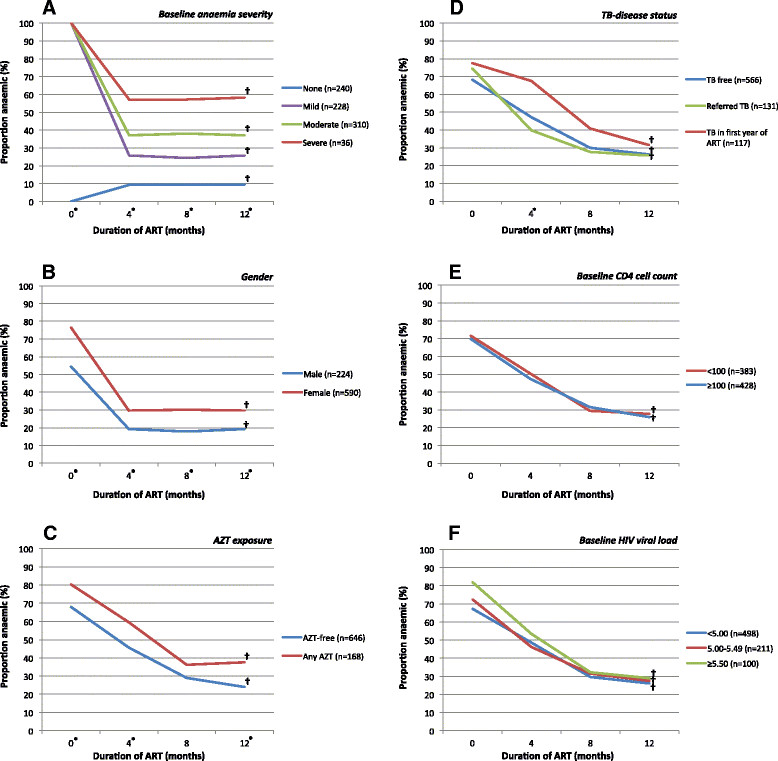
**Time-updated changes in the prevalence of anaemia (any severity) among patients receiving ART stratified by known or possible risk factors for anaemia A) baseline anaemia severity (classified according to WHO criteria) B) gender C) AZT exposure during first year of ART D) TB-disease status E) baseline CD4 cell count F) baseline HIV viral load.** *P < 0.05 for the difference in the proportion of patients with anaemia across each group at the given time interval using chi-squared test. †P < 0.05 for the difference in the proportion of patients with anaemia at baseline and those with anaemia after 12 months of ART for each individual variable using chi-squared test. Abbreviations: AZT, zidovudine; TB, tuberculosis.

During the first year of ART, the median haemoglobin level among all patients (n = 814) improved from 11.3 g/dL to 12.9 g/dL, representing a median improvement of 1.6 g/dL (p < 0.001). Significant improvements in haemoglobin levels and corresponding resolution of anaemia was observed during the first year of ART, irrespective of TB disease status, gender, AZT exposure, baseline anaemia severity, baseline CD4 count or baseline HIV viral load (Table 2 and Figure 3). While the greatest haemoglobin recovery was among those with moderate or severe anaemia at ART initiation, such patients were also much more likely to remain anaemic after 12 months of ART (Table 2 and Figure 3A). Despite significant increases in haemoglobin levels from baseline, females (Figure 3B) and those who had received an AZT-containing regimen (Figure 3C) were more likely to have anaemia 12 months after starting ART. Of note, the proportion of patients with anaemia and the median haemoglobin level improvement after 12 months of ART did not differ among those who had TB during the first year of ART compared to those who did not (p = 0.079 and p = 0.198, respectively) (Figure 3D).

**Table 2:** **Median haemoglobin improvement stratified by risk factors for anaemia (n = 814)**

	Overall median change in Hb from baseline to 12 months ART	P-value*	P-value**
**Overall**	+1.6	-	<0.001
**Gender**			
Male (n = 224)	+1.3	<0.001	<0.001
Female (n = 590)	+1.7		<0.001
**ART regimen**			
AZT-free throughout (n = 646)	+1.5	0.480	<0.001
Any AZT exposure (n = 168)	+1.7		<0.001
**Baseline anaemia severity**			
None (n = 240)	+0.4	<0.001	0.896
Mild (n = 228)	+1.5		<0.001
Moderate (n = 310)	+2.6		<0.001
Severe (n = 36)	+5.1		<0.001
**CD4 cell count (cells/μL)**			
<100 (n = 383)	+1.7	0.052	<0.001
≥100 (n = 428)	+1.4		<0.001
**Baseline HIV viral load (log copies/mL)**			
<5.00 (n = 498)	+1.4	<0.001	<0.001
5.00-5.49 (n = 211)	+1.8		<0.001
>5.50 (n = 100)	+2.1		<0.001
**TB disease status**			
TB free (n = 566)	+1.5	0.198	<0.001
Referred TB (n = 131)	+1.6		<0.001
TB in first year of ART (n = 117)	+1.7		<0.001

*P-value for difference in overall median haemoglobin change from baseline to 12 months after starting ART.

**P-value for difference between median haemoglobin level at baseline and median haemoglobin level after 12 months of ART.

Abbreviations: ART, antiretroviral therapy; TB, tuberculosis.

In a sensitivity analysis that included patients who died or were lost to follow-up during the first year of ART (n = 941) and who were assumed to have no haemoglobin improvement, the median improvement in haemoglobin levels after one year were slightly decreased (median overall improvement 1.3 g/dL, p < 0.001). However, overall and among all patient sub-groups, there was no difference in the improvement of haemoglobin levels (**data not shown**).

### Risk factors for anaemia after one year of ART

We undertook logistic regression analyses to determine factors independently associated with anaemia (any severity) after 12 months of ART. In univariable analysis, several risk factors met the pre-defined criteria for inclusion in the multivariable model, but notably TB in the first year of ART and CD4 cell count (baseline or time-updated) did not demonstrate associations with anaemia after 12 months of ART (Table 3). In the multivariable model, risk factors included greater severity of baseline anaemia, lower time-updated MCV and AZT exposure. Additional multivariable analyses demonstrated that the independent risk factors for both incident and persistent anaemia were identical to those identified among all patients, with the exception that baseline anaemia severity was not associated with incident anaemia.

**Table 3:** **Risk factors for anaemia after 12 months of ART use (n = 814)**

	Unadjusted OR (95% CI)	P-value	Adjusted OR (95% CI)	P-value
***Baseline characteristics***				
**Age (years)**				
<35	1.00	0.166		
≥35	1.01 (1.00-1.02)			
**Gender**				
Male	1.00	**0.002**	1.00	0.805
Female	1.78 (1.22-2.59)		1.06 (0.68-1.66)	
**WHO disease category**				
1/2	1.00			
3/4	1.02 (0.94-1.11)			
**CD4 category (cells/μL)**				
≥100	1.00	0.576		
<100	1.09 (0.80-1.49)			
**HIV viral load (log copies/mL)**				
<5.0	1.00	0.812		
5.0-5.49	1.07 (0.75-1.54)			
≥5.5	1.16 (0.72-1.86)			
**WHO anaemia severity**				
none	1.00	**<0.001**	1.00	**<0.001**
Mild	3.29 (1.95-5.55)		3.76 (2.14-6.62)	
Moderate	5.56 (3.42-9.06)		5.44 (3.14-9.40)	
severe	13.21 (6.00-29.10)		10.69 (4.52-25.28)	
**MCV (fL)**				
>100	1.00	0.093		
80-100	1.10 (0.63-1.92)			
<80	2.05 (0.96-4.35)			
***Time-updated characteristics***				
**AZT exposure in first year of ART**				
AZT-free throughout	1.00	**<0.001**	1.00	**0.009**
Any AZT exposure	1.90 (1.32-2.73)		1.74 (1.15-2.62)	
**CD4 category (cells/μL)**				
≥100	1.00	0.349		
<100	1.44 (0.68-3.01)			
**HIV viral load (copies/mL)**				
<1000	1.00	0.064	1.00	0.676
≥1000	1.91 (0.98-3.74)		1.18 (0.54-2.55)	
**MCV (fL)**				
>100	1.00	**<0.001**	1.00	**<0.001**
80-100	3.65 (2.50-5.32)		3.39 (2.23-5.17)	
<80	13.13 (2.70-63.93)		12.60 (2.12-74.80)	
**TB disease status**				
TB free in first year of ART	1.00	0.441		
Referred TB at ART initiation	0.95 (0.61-1.47)			
TB in first year of ART	1.31 (0.95-2.01)			

Abbreviations: AZT; zidovudine; MCV, mean corpuscular volume; TB, tuberculosis; WHO, World Health Organization.

## Discussion

Although anaemia was very common among patients starting ART in this South African cohort, this resolved in approximately two-thirds of patients during the first 12 months of ART. Significant haemoglobin recovery occurred regardless of prevalent and/or incident TB, gender, AZT exposure, baseline anaemia severity, CD4 cell count and HIV viral load measurements. These results suggest that ART is strongly associated with the resolution of HIV-related anaemia in the majority of patients without routine use of additional specific interventions.

More than 70% of patients initiating ART in our study had anaemia, consistent with other studies from sub-Saharan Africa [1]-[5]. While the prevalence of HIV-related anaemia will inevitably vary between settings, comparative studies have been made difficult by the use of non-standardized definitions for anaemia. Despite a WHO definition for anaemia that was proposed more than 45 years ago [53], several studies of HIV-related anaemia have used non-standardized definitions including simple haemoglobin concentration cut-offs for all patients that don’t take into account gender differences [1],[11],[40]-[42],[46],[54]. This may bias results towards lower prevalence estimates and underestimate the scope of the problem. Future research of HIV-related anaemia should use standardized definitions, such as the WHO proposed definition [49] to improve comparability of studies.

Receipt of ART for 12 months was associated with a 62% decrease in the overall prevalence of anaemia and a resolution of anaemia in 66% of patients with anaemia at ART initiation. Significant haemoglobin recovery was observed among all patients, including sub-groups stratified by risk factors for anaemia. Our findings are in agreement with several other studies from both low- and high-resource settings that demonstrate a strong, consistent benefit of ART for HIV-related anaemia [1],[14],[17],[35],[37]-[39],[41],[44],[45],[54],[55]. Importantly, less than 10% of patients receiving ART for 12 months developed new anaemia and this was predominantly mild in severity. For patients in whom anaemia is not life threatening and a blood transfusion is not required, these data suggest that use of ART alone is likely to result in the resolution of anaemia in the majority of patients.

Improvements in haemoglobin concentration did not differ between patients with prevalent and/or incident TB and those of patients who remained ‘TB-free’ throughout the first year of ART. TB is strongly associated with HIV-related anaemia in several settings [24]-[27]. This likely reflects the fact that both TB and HIV infection may contribute to downstream anaemia by acting upon a common pathway through the up-regulation of pro-inflammatory cytokines, especially IL-6, and a subsequent increase in hepcidin, which inhibits mucosal absorption of iron and favours sequestration of iron in bone marrow macrophages [18]. This results in inadequate iron availability for on-going erythropoiesis and eventually anaemia. The present study indicates that recovery of haemoglobin levels can be expected in patients with HIV-associated TB who receive ART for 12 months in combination with TB treatment for 6 months or more. Haemoglobin recovery in such patients is thus likely achieved through decreased immune activation.

Haemoglobin recovery among those exposed to an AZT-containing regimen was similar to those without exposure to AZT, consistent with other African-based studies [41],[45],[46],[55]. According to the 2013 WHO consolidated ART guidelines [56], AZT is recommended as part of alternative first line regimens for those in whom tenofovir/lamivudine/efavirenz is either contradicted or not available, and also as part of second line treatment. Reassuringly, when AZT is used or required, it appears that most African patients can be expected to nevertheless have significant recovery of haemoglobin concentrations. However, as AZT was an independent risk factor for anaemia after 12 months of ART [1],[37],[38],[42] a drug switch in those receiving AZT who remain anaemic during ART may nevertheless be required in a proportion of patients.

CD4 cell count and HIV viral load (either baseline or time-updated) were not independently associated with anaemia after 12 months of ART. Additionally, greater improvements in haemoglobin concentration were noted among those with very high baseline viral loads, consistent with other studies which have found large haemoglobin improvements among those with high viral load or advanced immunosuppression at baseline [37],[42] or among those achieving HIV viral suppression [55]. These data support the hypothesis that through a reduction in HIV viraemia and/or improvement of opportunistic infections and a subsequent decrease in immune activation, ART helps resolve anaemia of chronic disease [57]. Therefore, anaemia persisting after several months or years of ART may not be related to HIV disease, but due to other underlying causes.

Microcytosis (MCV < 80 fL) was uncommon, yet was a very strong independent predictor of anaemia after 12 months of ART, similar to previous studies [1],[2],[38],[41]. Microcytosis is suggestive of, but not diagnostic for, absolute iron deficiency and may also be caused by thalassaemia or anaemia of chronic disease, either with or without absolute iron deficiency [58]-[60]. Patients with iron-deficiency would not be expected to have resolution of anaemia with ART alone without increased dietary iron intake. Iron studies and additional inflammatory biomarkers were not available and we were therefore unable to further investigate the mechanisms that underpin HIV-related anaemia before and during ART. While ART with or without anti-TB treatment may result in a reduction of cytokine-associated cachexia [61] with resultant improved nutritional status, iron studies may be warranted in patients with moderate or severe anaemia prior to ART initiation or in those who remain anaemic despite ART, in order to identify patients that may benefit from iron supplementation.

While nearly three-quarters of patients had normal haemoglobin levels after receipt of ART for 12 months, an important minority (27%) remained anaemic. Although only 8% of all patients had moderate or severe anaemia after 12 months ART, even mild anaemia can be associated with decreased quality of life and increased mortality risk [6]-[9],[17],[62]. Because HIV-related anaemia is complex and likely multifactorial is not surprising that some patients may require multiple interventions. Therefore, elucidating additional causes of anaemia for those with persistent anaemia during ART is important for determining which interventions are appropriate and when they should be considered during ART. Given that those who had moderate or severe anaemia at ART initiation were more likely to have persistent anaemia during ART and also constituted two-thirds of those who died during the first year of ART, such patients should be prioritized for additional investigations +/− adjunctive interventions either when initiating ART, or very early during ART. Interventions may include routine microbiological investigation for TB [24], nutritional supplements, additional laboratory investigation for and treatment of concomitant diseases and opportunistic infections, changes to medications known to be associated with anaemia (including trimethoprim-sulphamethoxazole and AZT) and should include more frequent patient follow-up to determine when a patient may require hospitalisation [58].

Study strengths include that this was an analysis of prospectively collected data from a large well-characterized cohort. This study expands previous knowledge by reporting haemoglobin recovery at multiple time points during the first year of ART, using a standardised definition for anaemia, and being undertaken in a very high TB incidence setting. While anaemia interventions were not implemented routinely, data was not available regarding treatment of anaemia for patients and this may have contributed to haemoglobin recovery in some individuals. Pregnancy status was not available during ART follow-up, which may have slightly overestimated the prevalence of baseline anaemia, contributed to incident or persistent anaemia during ART and some pregnancy-related anaemia may have otherwise resolved without ART. However, in previous studies conducted at the same study clinic, the proportion of females initiating ART who were pregnant was small and ranged from 4 to 14% [63],[64]. Data regarding contributing factors to anaemia such as toxicity from trimethoprim-sulphamethoxazole, acute blood loss, other opportunistic infections and nutritional deficiencies, as well as attributable causes of death, were not available and thus could not be further investigated. However, this study was done under routine programme conditions, which improves generalizability of findings. By including only patients with haemoglobin results available at ART and 12 months, haemoglobin recovery associated with ART may have been overestimated by excluding some patients who died or lost-to-follow-up and would be expected to have poor haemoglobin recovery. However, sensitivity analyses including such patients still demonstrated strong evidence of haemoglobin recovery both overall and within each sub-group of patients. Additionally, patients known to be alive, but without 12-month haemoglobin results available, had similar baseline characteristics and haemoglobin recovery compared to those included in the final analysis suggesting that their exclusion likely did not affect the findings.

## Conclusion

In conclusion, despite very advanced immunodeficiency and a high prevalence of anaemia at ART initiation, approximately three-quarters of patients had normal haemoglobin levels after 12 months of ART, including two-thirds of patients with baseline anaemia. ART was associated with significant haemoglobin recovery irrespective of gender, ART regimen, prevalent and/or incident TB or CD4 cell count. A large majority of patients receiving ART will have resolution of anaemia without additional specific interventions and therefore, ART should be considered the primary intervention for HIV-related anaemia in sub-Saharan Africa. However, for the one-quarter of patients who do not achieve normalization of haemoglobin levels during the first year of ART, additional investigations +/− interventions may be required.

## Authors’ original submitted files for images

Below are the links to the authors’ original submitted files for images. Authors’ original file for figure 1 Authors’ original file for figure 2 Authors’ original file for figure 3
