# Supplementary figures and images for: Resolution of anaemia in a cohort of HIV-infected patients with a high prevalence and incidence of tuberculosis receiving antiretroviral therapy in South Africa
Source: BMC Infect Dis. 2014 Dec 21;14:3860. doi: 10.1186/s12879-014-0702-1 (PMC4300078; doi:10.1186/s12879-014-0702-1)

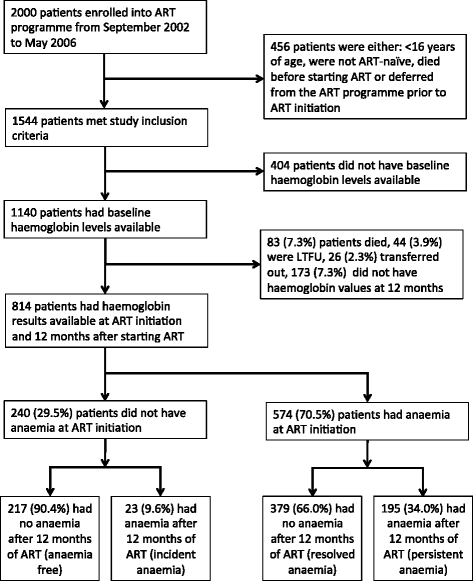

Supplement: Supplementary file 1 — Authors’ original file for figure 1 [file 12879_2014_702_MOESM1_ESM.gif]

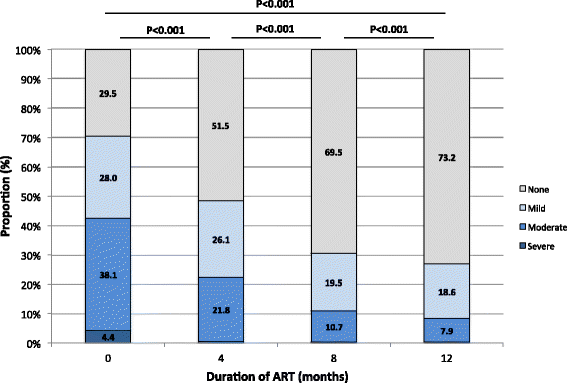

Supplement: Supplementary file 2 — Authors’ original file for figure 2 [file 12879_2014_702_MOESM2_ESM.gif]

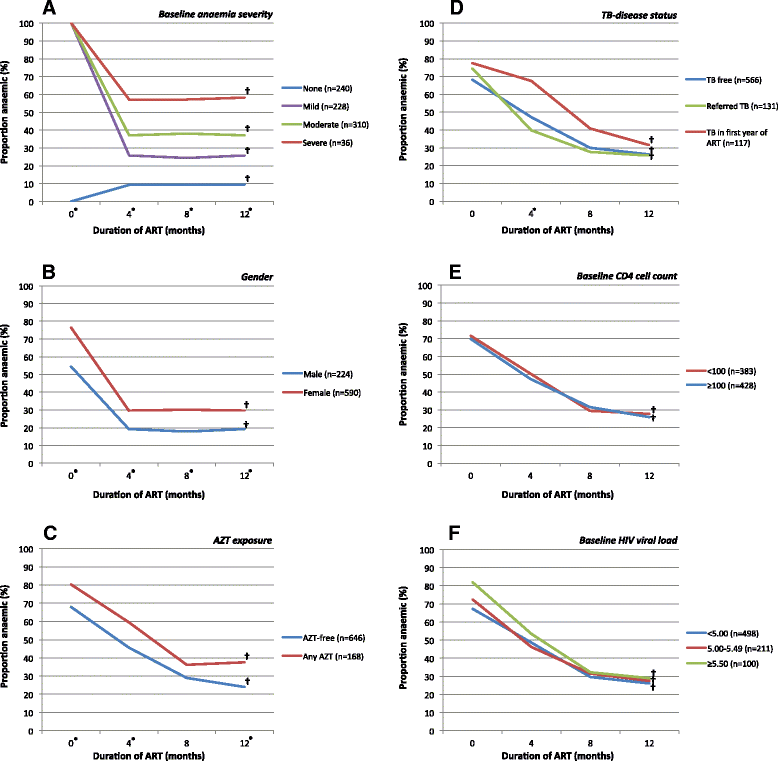

Supplement: Supplementary file 3 — Authors’ original file for figure 3 [file 12879_2014_702_MOESM3_ESM.gif]
